# Evaluation of the performance of three pulse oximeters at different probe positions in awake rabbits

**DOI:** 10.1371/journal.pone.0323044

**Published:** 2025-05-15

**Authors:** Giulia Maria De Benedictis, Barbara Contiero, Daniele Bovo, Vincenzo De Rosa, Martina Cardinali, Francesca Zanusso

**Affiliations:** 1 Department of Animal Medicine, Production and Health, University of Padova, Legnaro, Italy; 2 Department of Small Animal Internal Medicine, Southern Counties Veterinary Specialists, Ringwood, United Kingdom; Veterinary College and Research Institute, Orathanadu, INDIA

## Abstract

In rabbits, pulse oximetry plays a crucial role in monitoring oxygen saturation (SpO_2_) and pulse rate (PR), but it can be challenging due to their small size, variable anatomy, limited measurement sites, and potential motion artifacts during measurements. This prospective randomized study aimed to assess the reliability of three pulse oximeters on the forelimb and tail of fifty awake, healthy European rabbits. Two pulse oximeters, the Masimo Rad-5 (device 1) and the Edan VE-H100B, were tested, with the latter using both a Y-clip (device 2) and the Nellcor OxiMax adhesive finger sensor (device 3). Reliable values of SpO_2_ and PR were considered ≥ 95% and ≥ 151 bpm, respectively, which are normal values in a healthy rabbit. Success rates of the devices were calculated as the ratio of reliable to unreliable/missing values and compared using χ^2^ tests. Correlation and agreement between PR and clinically measured heart rate (cHR) were assessed using the Pearson correlation coefficient (r) and Bland-Altman analysis, respectively. Success rates differed significantly among devices (p < 0.001). The SpO_2_ success rates were 46%, 95.6%, and 94.4% for devices 1, 2, and 3, respectively. The PR success rates were 54.6%, 63%, and 72.8% for devices 1, 2, and 3, respectively. Success rates were higher on the forelimb (>66%) than on the tail (>26%) across devices. Correlation between PR and cHR was stronger on forelimb (r > 0.80) than on tail (r < 0.70). Agreement between PR and cHR was similar among devices, with a mean bias ranging from -6.6 to 6.7 bpm, and limits of agreement from -33.8 to 41.5 bpm. The Edan devices showed superior ability to detect reliable values of SpO_2_ and PR compared to the Masimo in rabbits. The forelimb appears to be a more reliable site for pulse oximetry in rabbits than the tail.

## Introduction

Pulse oximetry has become the point-of-care tool of choice in veterinary medicine in recent years. This non-invasive and easily accessible tool evaluates the oxygenation status of patients by estimating the percentage of arterial hemoglobin saturated with oxygen (SpO_2_). Pulse oximetry offers multiple potential advantages, including cost-effectiveness, ease of use, and continuous or long-term monitoring without the need for arterial catheterization or repeated arterial puncture. Moreover, by simultaneously measuring both SpO_2_ and pulse rate (PR), it provides real-time information related to both the cardiovascular and pulmonary systems of veterinary patients [1].

Therefore, the use of pulse oximetry is strongly recommended to improve the safety of anesthesia in veterinary patients [2]. Its utility is crucial both in pre-anesthetic evaluation and during anesthesia in rabbits, which have one of the highest peri-anaesthetic mortality rates of any domestic species [3–5]. Pulse oximetry can help detect subclinical pathology in awake patients, such as respiratory impairment, which may not be evident during clinical pre-anaesthetic evaluation. In anesthetized rabbits, pulse oximetry can monitor cardiorespiratory depression, a common effect of most anesthetics that can often lead to hypoxemia [3].

However, pulse oximeters used in veterinary medicine are often designed for humans and can be inadequate for rabbits, which have higher PR values compared to humans and have limited anatomical sites for the pulse oximeter probe.

Although proven to provide accurate monitoring even in conditions of motion and low perfusion, Masimo Rad-5, which is a commonly used pulse oximeter in veterinary medicine, can only detect PR up to 240 bpm [6]. In contrast, the pulse oximeter Edan VE-H100B, designed for veterinary patients, can measure PR up to 350 bpm, but the manufacturer warns that motion may affect the accuracy of the measurements [7].

Another challenge in the use of pulse oximetry in rabbits is related to probe position. In veterinary medicine, the tongue is the standard anatomical location for pulse oximeters. However, rabbits have very small tongues, and the conventional Y-clip pulse oximeter probe can be difficult to position. Furthermore, a rabbit tongue is difficult to extend, resulting in frequent measurement failures and probe displacement. Additionally, in dental or oral procedures, which are commonplace in this species, the tongue is entirely inaccessible. If an endotracheal tube or a laryngeal mask is used, the space occupied by the device can prevent correct positioning of the probe on the tongue [8]. Furthermore, the tongue cannot be used for this purpose in awake rabbits. The ear may represent an alternative probe location for the Y-clip, but excessive compression of the auricular vasculature by the clamp holding the probe may result in poor signal [9].

The limbs and tail are other alternative probe locations [10,11]. These locations can be too small for the Y-clip in some rabbits anyway. Other types of probes, such as the Nellcor Oxi Max adhesive finger sensor, which is an adhesive tape, can be wrapped around the limb, as made in humans around a finger [12].

Given the critical importance and the challenges in the application of pulse oximetry in rabbits, it is essential to investigate the reliability of available tools for measuring SpO_2_ and PR in this species. A comparative study of different pulse oximeters in rabbits, similar to those conducted in other animal species [13–16], may be beneficial.

While previous studies have evaluated the performance of pulse oximetry in anesthetized rabbits [17,18], data on its use in awake animals remains scarce. Furthermore, although the forelimb has been investigated as a probe placement site, no studies have evaluated the effectiveness of pulse oximetry on the rabbit tail. This site may provide a convenient and accessible alternative for clinical procedures. Additionally, motion is known to interfere with SpO₂ readings, yet little is known about how movement affects pulse oximeter performance in rabbits. Moreover, no studies have specifically examined the ability of these devices to provide reliable pulse rate measurements in this species.

Therefore, the primary objective of this study was to evaluate the ability of three different pulse oximetry devices (Masimo Rad-5 equipped with a Y-clip, Edan VE-H100B equipped with a Y-clip, and the Edan VE-H100B equipped with a Nellcor Oxi Max adhesive finger sensor) to provide reliable values of SpO_2_ and PR in awake rabbits when placed on two distinct alternative anatomical sites (forelimb and tail). The second objective was to investigate the accuracy of PR measured by each device at each site.

We hypothesized that the Edan VE-H100B equipped with a Nellcor Oxi Max adhesive finger sensor would provide more reliable values of SpO_2_ and PR in awake rabbits compared to the other devices.

## Materials and methods

This prospective randomized study was conducted under the ethical approval from the Animal Welfare Body of the University of Padova (OPBA approval number 49/2022) and in accordance with the ARRIVE guidelines [19]. Clients were informed of the study aims, provided informed consent and signed a university-approved informed consent form.

### Animals

A total of fifty commercial hybrid European rabbits (*Oryctolagus cuniculus*) from the same breeding facilities were enrolled in the study when brought to the Veterinary Teaching Hospital of the University of Padova for routine vaccination and examination. Animals of both sexes, deemed healthy on the basis of a complete physical examination, were included in the study if they had a body condition score (BCS) [20] equal to or greater than 3/5, were aged between 9 months and 2 years, were not pregnant, and had a white tail and at least one white forelimb. They also had to be docile and accustomed to human handling. This was assessed based on their behavior during routine handling in the clinic, ensuring that they tolerated gentle restraint for 3 minutes without showing signs of distress or aggression. Animals were excluded if they showed clinical signs of illness or distress, had a history of previous disease, or were uncooperative with manipulation. During the study, all rabbits were awake and only gently restrained in sternal recumbency on the examination table throughout the clinical trial. To ensure their comfort and minimize stress, they gently restrained around the body and hind limbs.

### Devices

**Fig 1:**
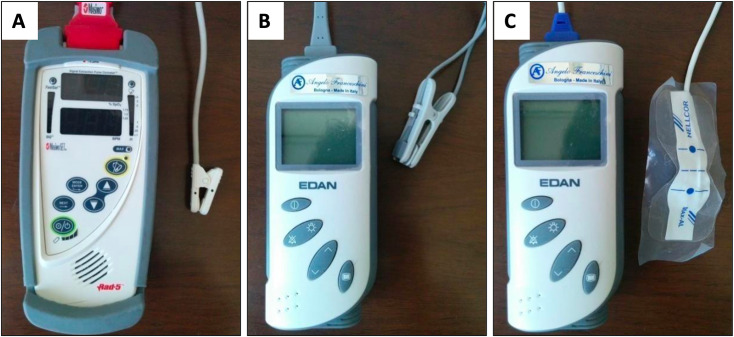
The three pulse oximeters tested in the study on rabbits, applied to the forelimb and tail. (A) Masimo Rad-5 with a Y-clip sensor (device 1), (B) Edan VE-H100B with a Y-clip sensor (device 2), and (C) Edan VE-H100B with a Nellcor OxiMax adhesive finger sensor (device 3).

Three different pulse oximeters were tested: the Masimo Rad-5 (Masimo Corporation, Irvine CA, USA), equipped with a Y-clip (device 1), the Edan VE-H100B (Edan Instruments, Inc., Shenzhen, China) equipped with a Y-clip (device 2), and the Edan VE-H100B equipped with a Nellcor Oxi Max adhesive finger sensor (device 3) (Fig 1).

### Measurements

**Fig 2:**
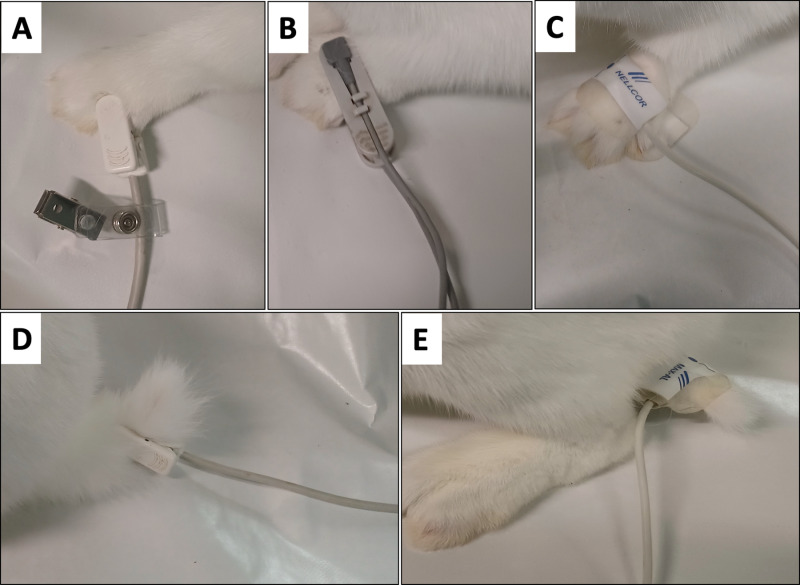
Application of pulse oximeter probes to the forelimb and tail of a rabbit. (A) Masimo Rad-5 Y-clip sensor (device 1) applied to the forelimb, (B) Edan VE-H100B Y-clip sensor (device 2) applied to the forelimb, (C) Edan VE-H100B Nellcor OxiMax adhesive finger sensor (device 3) applied to the forelimb, (D) Masimo Rad-5 Y-clip sensor (device 1) applied to the tail, and (E) Edan VE-H100B Nellcor OxiMax adhesive finger sensor (device 3) to the tail.

One of the three pulse-oximeters was alternately placed on the tail and on the right forelimb in randomized order (Fig 2). The location and positioning of the probe was performed as described for awake rabbits [4,21].

Each device was placed on each animal at each site for 2 minutes to obtain a total of 30 measurements of SpO_2_ and PR for each rabbit. During these 2 minutes, the device was left in place for 45 seconds to allow the reading to stabilize, as done by Dörfelt *et al.* [16] in cats, where they waited one minute. However, in awake rabbits, 45 seconds was deemed sufficient to achieve a stable signal, based on the authors’ experience. After this stabilization period, a series of 5 SpO_2_ and 5 PR measurements were recorded at 15-second intervals. During the SpO_2_ measurements, ambient light was reduced to prevent signal interference and kept consistent for all rabbits. Evaluations were conducted in the same room, with no other animals present. Moreover, clinical heart rate (cHR) was measured three times from each animal by the same veterinarian (DB) during placements of the devices. These clinical heart rate measurements were taken at intervals of at least 15 seconds through cardiac auscultation.

If the animal moved during the measurements, potentially affecting the accuracy of the measured parameters, the session was paused, and the rabbit was allowed to rest for at least 15 minutes before starting a new session.

Values of SpO_2_ and PR measured by the devices were classified as *missing*, *reliable* or *non-reliable*, based on physiological parameters typically reported in literature for rabbits [4]. In clinically healthy rabbits a normal oxygenation status is expected with SpO_2_ greater than 95% and heart rate greater than 150 bpm. When the devices failed to produce any number, the measurements were considered missing (*missing*). Values of SpO_2_ < 95% and PR < 151 bpm were considered clinically unreasonable (*non-reliable*). Measurements were considered *reliable* when SpO_2_ ≥ 95% and PR ≥ 151 bpm.

### Statistical analysis

With respect to the SpO_2_ values, a sample size of 211 measurements for each pulse oximeter at each location was calculated to detect a 2% difference among devices with a standard deviation of 3.5%, with an alpha of 0.05 and a power of 0.9. The 2% difference is considered the smallest possible difference to validate the performance of a standard pulse oximeter in human medicine [22].

For PR, an estimated sample size of 123 measurements for each pulse oximeter at each position was required to detect a significant difference of 5 bpm among devices when considering a standard deviation of 8 bpm, with an alpha of 0.05 and a power of 0.9.

Considering a drop rate of 18%, a sample size of 249 measurements for each device at each location was considered. Therefore, aiming to collect 5 measurements from each rabbit for each device at each location, we decided to include a total of 50 animals in the study.

The Shapiro-Wilk test was used to assess the normal distribution of values. Data were presented as mean ± SD for normally distributed variables or as median (min-max) for those not following a normal distribution. *Reliable* values of SpO_2_ and PR were compared among the devices, and between probe location using ANOVA (with the effect of animal included in the model as random) or Kruskal-Wallis test as appropriate.

*Success rates* for SpO_2_ and PR were then calculated as percentages for each device at each location. *Success rates* included *reliable* measurements out of total measurements. *Success rates* were compared among devices and location via the χ^2^ test.

Moreover, the accuracy of *reliable* PR values measured by each device at each site was evaluated. Since PR was measured 5 times by each device at each location and cHR was assessed three times, not always simultaneously with PR, the mean of the 5 measurements of PR value was compared with the mean of the 3 measurements of cHR (mcHR) for each animal. The correlation between mcHR and r*eliable* values of PR was assessed using the Pearson correlation coefficient. The agreement between mcHR and *reliable* values of PR was assessed by Bland–Altman analysis for each device at each site.

All statistical analysis were performed using SAS (Inst. Inc., Cary, NC, USA) and XLSTAT for Excel (https://www.xlstat.com). A significance level of p* *< 0.05 was used to determine statistical significance.

## Results

The rabbits included in the study were 20 males and 30 females aged 1.03 ± 0.12 years and weighing 1.00 ± 0.07 kg. Data were collected from September 2022 and December 2023.

A total of 1500 measurements of SpO_2_ and PR were obtained, with 250 measurements for each parameter taken for each device and probe location.

For SpO_2_, a total of 1255 measurements were *reliable*, 150 were *missing*, and 95 were *non-reliable* (Table 1). *Missing* measurements were more frequent on the forelimb (n = 90) than on the tail (n = 60), while *non-reliable* measurements were more frequent on the tail (n = 77) than on the forelimb (n = 18).

**Table 1:** Number of *reliable*, *missing* and *non-reliable* measurements of hemoglobin oxygen saturation (SpO_2_) and pulse rate (PR) using the Masimo Rad-5 equipped with a Y-clip (device 1), the Edan VE-H100B equipped with a Y-clip (device 2), and the Edan VE-H100B equipped with Nellcor Oxi Max adhesive finger sensor (device 3) placed on the forelimb and tail.

Parameter	Location	Measurement classification	Device 1 (n)	Device 2 (n)	Device 3 (n)
**SpO**_**2**_	Overall	*reliable*	230	478	472
		*missing*	215	20	20
		*non-reliable*	55	2	8
	Forelimb	*reliable*	165	240	237
		*missing*	70	10	10
		*non-reliable*	15	0	3
	Tail	*reliable*	65	238	235
		*missing*	145	10	10
		*non-reliable*	40	2	5
**PR**	Overall	*reliable*	273	315	364
		*missing*	215	0	10
		*non-reliable*	12	185	126
	Forelimb	*reliable*	168	195	212
		*missing*	70	0	0
		*non-reliable*	12	55	38
	Tail	*reliable*	105	120	152
		*missing*	145	0	10
		*non-reliable*	0	130	88

Out of the 1500 PR measurements, 952 measurements were *reliable*, 225 were *missing*, and 323 were *non-reliable* (Table 1). *Missing* measurements were more frequent on the tail (n = 155) than on the forelimb (n = 70). Additionally, *non-reliable* measurements were more frequent on the tail (n = 218) than on the forelimb (n = 105). Among the devices, device 2 had the highest number of *reliable* values of SpO_2_, on both the forelimb and on the tail, as well as the highest number of *reliable* PR values on the forelimb. In contrast, device 1 presented the lowest number of *reliable* values for both SpO_2_ and PR. Additionally, device 1 had the highest number of *missing* values for both SpO_2_ and PR, both on the forelimb and on the tail. Device 1 also had the highest number of *non-reliable* measurements for SpO_2_, while presenting the lowest number of *non-reliable* measurements for PR. On the other hand, device 2 had the lowest number of *non-reliable* measurements for SpO_2_ but had the highest number of *non-reliable* measurements for PR. When considering both SpO_2_ and PR, device 1 failed to detect SpO_2_ whenever it failed to detect PR. Device 2 identified *non-reliable* PR values for all 10 *missing* forelimb SpO_2_ measurements and 8 out of 10 *missing* tail SpO_2_ measurements. Device 3 provided *reliable* PR values for all 10 *missing* forelimb SpO_2_ measurements but did not detect PR values for any of the 10 *missing* tail SpO_2_ measurements.

In terms of animals, device 1 failed to detect SpO_2_ and PR in 14 rabbits on the forelimb and in 8 animals on the tail. Both device 2 and device 3 failed to detect SpO_2_ in the same two rabbits on the forelimb and on the tail.

*Reliable* SpO_2_ and PR values for each device at each location are shown in Table 2. No statistically significant differences in SpO_2_ and PR values were found between the devices or probe location using Kruskal-Wallis test.

**Table 2:** *Reliable* values of hemoglobin oxygen saturation (SpO_2_) and pulse rate (PR) using the Masimo Rad-5 with a Y-clip (device 1), the Edan VE-H100B with a Y-clip (device 2), and the Edan VE-H100B with Nellcor Oxi Max adhesive finger sensor (device 3) on the forelimb and on the tail. The data were presented as median (min-max).

Parameter	Location	Device 1	Device 2	Device 3
**SpO**_**2**_ **(%)**	Forelimb	98 (95-100)	100 (99-100)	100 (96-100)
	Tail	98 (95-100)	100 (96-100)	100 (95-100)
**PR (bpm)**	Forelimb	207 (160-240)	214 (151-254)	212 (163-340)
	Tail	207 (159-236)	200 (163-238)	225 (162-259)

The *success rates* of all devices were higher on the forelimb compared to the tail, ranging from 66% to 96% for the forelimb, and from 26% to 94% for the tail (Table 3). A statistically significant difference was found among devices in the *success rate* of SpO_2_ and of PR at all positions (p < 0.0001). Device 1 had a lower *success rate* for both SpO_2_ and PR compared to the other devices.

**Table 3:** *Success rates* of hemoglobin oxygen saturation (SpO_2_) and pulse rate (PR) using the Masimo Rad-5 with a Y-clip (device 1), the Edan VE-H100B with a Y-clip (device 2), and the Edan VE-H100B with Nellcor Oxi Max adhesive finger sensor (device 3) on the forelimb and tail.

Parameter	Location	Device 1	Device 2	Device 3	p-value *
**SpO**_**2**_	Overall	46.0%	95.6%	94.4%	<0.001
	Forelimb	66.0%	96.0%	94.8%	<0.001
	Tail	26.0%	95.2%	94.0%	<0.001
**PR**	Overall	54.6%	63.0%	72.8%	<0.001
	Forelimb	67.2%	78.0%	84.8%	<0.001
	Tail	42.0%	48.0%	60.8%	<0.001

* Compared by the χ^2^ test

The cHR was obtained by auscultation of the heart in all animals and was recorded as 204 (151–260) bpm. Thus, no rabbit had cHR below 151 bpm. The correlation between mcHR and *reliable* PR values was statistically significant for all positions and devices (Table 4). Notably, the correlation was higher on the forelimb (r > 0.80) than on the tail (r < 0.70) for all devices. Specifically, on the forelimb the correlation was very strong for all devices. Conversely, on the tail the correlation was strong for device 1 (r = 0.68), and for device 2 (r = 0.70), and very weak for device 3 (r = 0.19).

**Table 4:** Pearson correlation coefficient (r) between clinical heart rate (cHR) and *reliable* pulse rate (PR) values using the Masimo Rad-5 with a Y-clip (device 1), the Edan VE-H100B with a Y-clip (device 2), and the Edan VE-H100B with Nellcor Oxi Max adhesive finger sensor (device 3) on the forelimb and tail.

Location	Device	r	p-value
Forelimb	1	0.80	<0.001
	2	0.82	<0.001
	3	0.85	<0.001
Tail	1	0.68	<0.001
	2	0.70	<0.001
	3	0.19	<0.001

**Fig 3:**
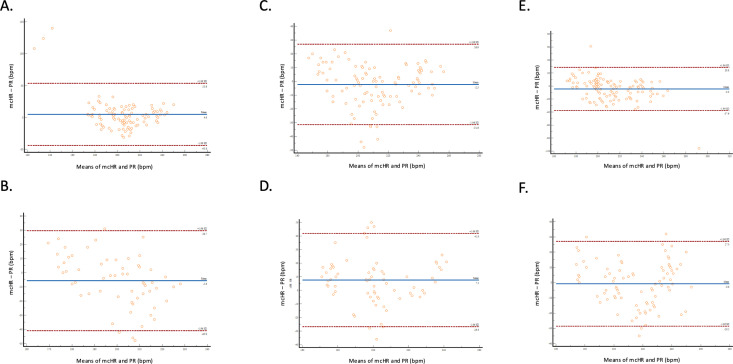
Limits of agreement (Bland–Altman) plot showing differences between mean clinical heart rate (mcHR) and *reliable* pulse rate (PR) values in 50 rabbits. Differences are shown using the Masimo Rad-5 with a Y-clip (device 1) on the forelimb (A) and on the tail (B); using the Edan VE-H100B with a Y-clip (device 2) on the forelimb (C) and on the tail (D); and using the Edan VE-H100B with Nellcor OxiMax adhesive finger sensor (device 3), on the forelimb (E) and on the tail (F).

Agreement between mcHR and *reliable* PR values was assessed by Bland–Altman analysis and showed a smaller mean bias on the forelimb than on the tail for device 1 and 2 (Table 5, Fig 3). The smallest limit of agreement was obtained for device 3 on the forelimb (-26.63 to 18.78 bpm).

**Table 5:** Mean bias with 95% limits of agreement, assessed using Bland–Altman analysis, between mean clinical heart rate (mcHR) and *reliable* pulse rate (bpm) values measured by the Masimo Rad-5 with a Y-clip (device 1), the Edan VE-H100B with a Y-clip (device 2), and the Edan VE-H100B with Nellcor Oxi Max adhesive finger sensor (device 3), in 50 rabbits on the forelimb and tail.

Location	Device	Mean bias (95% confidence interval)	Lower limit of agreement. (95% confidence interval)	Upper limit of agreement (95% confidence interval)
Forelimb	1	3.86 (-2.64, 10.36)	-33.81 (-45.02, -22.59)	41.52 (30.30, 52.73)
	2	-3.23 (-7.20, 0.73)	-27.86 (-34.69, -21.03)	21.39 (14.55, 28.22)
	3	-3.92 (-7.45, -0.40)	-26.63 (-32.70, -20.57)	18.78 (12.71, 24.85)
Tail	1	-6.55 (-12.75, -0.34)	-33.27 (-44.05, -22.49)	20.18 (9.40, 30.95)
	2	6.65 (-0.03, 13.34)	-24.37 (-35.95, -12.78)	37.68 (26.09, 49.26)
	3	-0.26 (-4.86, 4.33)	-24.83 (-32.77, -16.88)	24.30 (16.36, 32.25)

## Discussion

This study provided a comprehensive examination of the performance of two of the most commonly used pulse oximeters in veterinary medicine, the Masimo Rad-5 and the Edan VE-H100B, in a large population of rabbit. The evaluation of pulse oximeters in the accurate detection of both SpO_2_ and pulse rate in these animals is of significant importance, as the validation of pulse oximetry devices remains lacking despite recommendations for their use, particularly during anesthesia [23]. Rabbits are among the species with the highest anesthesia‐related mortality, with more than 60% of anesthesia-related deaths in rabbits occurring after completion of anesthesia [24]. Thus, monitoring cardiorespiratory function in rabbits is crucial, especially during the perioperative period when the tongue may not be available, and alternative locations for the pulse oximeter probe should be considered. This study was useful to partially fill the knowledge gap regarding the ability of different pulse oximeters to detect SpO_2_ and pulse rate in two alternative locations, the forelimb and the tail, which are also suitable in awake rabbits. While previous studies [17,18] have evaluated the performance of pulse oximetry in the forelimb of anesthetized rabbits, to our knowledge, no study has assessed the effectiveness of pulse oximetry on the rabbit tail. We believe that this site could be easily accessible for various clinical procedures, and provide an additional monitoring option, particularly in settings where access to other anatomical sites may be limited. Furthermore, this study allowed for the evaluation of pulse oximetry performance in awake rabbits. Since motion is known to interfere with SpO₂ readings, it is crucial to assess the accuracy of the device under these conditions. Additionally, there are no existing studies that have evaluated the ability of pulse oximeters to reliably measure pulse rate in rabbits. This study provided valuable insights into this aspect as well.

The results of this study suggest that the Edan VE-H100B has a greater ability to detect reliable values of SpO_2_ and pulse rate compared to the Masimo Rad-5 in awake rabbits. Thus, the Edan VE-H100B may improve patient safety during clinical evaluations and perioperative monitoring in rabbits. Notably, the success rate for SpO_2_ was very high with the Edan VE-H100B, whether equipped with a Y-clip (95.6%) or Nellcor Oxi Max adhesive finger sensor (94.4%). Therefore, the Edan VE-H100B allows for more effective monitoring of oxygenation status in awake rabbits, ensuring more reliable measurements in clinical settings. This high success rate supports the use of pulse oximetry as a viable monitoring tool in rabbits, a species in which assessment of oxygenation status is not always easy. The observed differences in device performance could be attributed to several factors, including variations in probe pressure, sensor design, and local blood flow dynamics at the measurement sites. The Masimo Rad-5, which had a lower success rate, may have been more affected by suboptimal sensor placement or insufficient tissue perfusion. Additionally, the stronger pressure applied by the Y-clip compared to the Nellcor Oxi Max finger adhesive sensor may have contributed to signal loss, particularly on the tail, where vascular structures are more delicate and prone to compression-induced occlusion. Regarding the performance of different devices, these findings differ from a study in anesthetized cats [16], where the Masimo Rad-5 showed a lower failure rate compared to the Edan H100N. However, it is important to note that the Edan H100N differs from the Edan VE-H100B used in our study, as the latter is specifically designed for veterinary patients and can detect high pulse rates up to 350 bpm. Therefore, it is possible that the different results in our study on rabbit were influenced by the different device used. In our study, the lower success rate of the Masimo device was primarily due to its failure to detect SpO_2_ and PR values, rather than its potential to provide unreliable readings. Therefore, although the Masimo Rad-5 appears to produce reliable values less frequently than the Edan VE-H100B, the likelihood of recording an inaccurate value is low. This finding is further supported by the general high agreement between the pulse rate measured by the Masimo Rad-5 and the clinical heart rate. Despite its accuracy, the Masimo Rad-5 had a very low overall success rate for both SpO_2_ (46%) and pulse rate (54.6%). The low success rate of this device makes it clinically unsuitable for awake rabbits, especially when the Y-clip was placed on the tail, where the success rate dropped to 42% in our study. These results suggest that the Masimo Rad-5 may have limited applicability in clinical settings that require continuous or real-time monitoring of SpO₂ and pulse rate. Given the importance of accurate and consistent measurements for assessing cardiovascular and respiratory status, the low success rate of this device could lead to missed hypoxemic events or delayed clinical interventions. This limitation is particularly relevant in anesthesia monitoring, where accurate and frequent measurements are essential. Consequently, the more reliable Edan VE-H100B may be more suitable for clinical use in rabbits. However, it should be noted that the two devices do not have the same cost, and the Y-clip may be easier and quicker to apply to the rabbit. On the other hand, the adhesive probe might take longer to apply to the forelimb or tail compared to the Y-clip, which could be a source of stress for the animal.

The high success rate of the Edan VE-H100B in this study supports its clinical use as a pulse oximeter for measuring SpO_2_ in awake patients from both the forelimb and the tail. These anatomical sites may also be easily accessible in awake patients where the assessment of oxygenation status is also important. Device failures may have been influenced by technical factors such as improper probe positioning, excessive motion artifacts, and variable local perfusion. Motion is particularly relevant in awake patients, as even minimal movement can introduce signal disturbances. Additionally, differences in individual vascular anatomy and skin pigmentation may have played a role in the variable success rates observed between different subjects. In our study, the Edan VE-H100B failed to detect a reliable SpO_2_ value in only 5.6% of measurements. In comparison, a study evaluating five pulse oximeters in dogs, cats, and horses with probes placed at five sites reported a higher failure rate of SpO_2_ measurement, up to 60% [25]. The high success rate in our study could also be attributed to the specific selection of patients, all of whom had a white tail and at least one white forelimb. This choice was made to minimize potential interference from pigmentation, which has been reported to affect the accuracy of pulse oximeters in animals [26]. However, it is important to recognize that there is a variation in coat color among rabbits, including agouti, chinchilla, and albino patterns. Under clinical conditions, where animals of different colors are presented, the performance of pulse oximeters may differ. Future studies should address this limitation by including rabbits with a wider range of coat colors to better assess the effect of pigmentation on SpO₂ measurements and ensure that the results are more representative of real-world clinical scenarios.

In this study, when the Edan VE-H100B failed, it primarily failed to detect SpO_2_ values rather than displaying unreliable SpO_2_ values. Conversely, for pulse rate, the Edan VE-H100B missed only 10 out of 1000 measurements but provided 301 non-reliable measurements. This indicates that the Edan VE-H100B may erroneously detect a low pulse rate, below 150 bpm. However, when the detected rate is above 151 bpm, the value appears to be quite accurate, as the overall agreement between pulse rate values recorded by this device and clinical heart rate values is good, with 95% limits of agreement ranging from -33–37 bpm. From a clinical perspective, these results suggest that while the Edan VE-H100B is generally reliable, caution is needed when interpreting pulse rate values below 150 bpm, as they may be underestimated. This could be particularly relevant in scenarios requiring accurate monitoring of cardiovascular function, such as anesthesia, where an undetected bradycardia could impact clinical decision making. Additionally, the tendency of the device to fail to detect SpO₂ rather than provide inaccurate values implies that a lack of reading should not be assumed to indicate normoxemia but rather a problem with probe placement or signal acquisition. Clinicians should be aware of these limitations and consider using complementary monitoring methods when necessary to ensure accurate patient assessment. Regarding the variability of pulse rate values, a study comparing the accuracy of a third-generation pulse oximeter (Dolphin Voyager) with a first-generation device (Nellcor N-180) in anesthetized dogs found a bias of only 1.4 bpm, indicating that pulse rate monitoring with these devices is highly reliable [13]. The slight inaccuracies of the Edan VE-H100B device in measuring pulse rate in this study may be attributed to slight movements of the awake patients. According to the manufacturer, the performance of the device can be affected by motion [7]. Spontaneous patient movement is known to interfere with pulse rate detection. Even brief shifts in sensor position can introduce significant artifacts in optical measurements made by pulse oximeters. Moreover, if these artifacts resemble a heartbeat, the device may have difficulty distinguishing between motion-induced pulsations and genuine arterial pulsations, resulting in inaccurate pulse readings. The main source of motion artifacts in pulse oximetry is typically due to changes in light transmission caused by sensor movement [27]. In our study, rabbits were gently restrained by supporting the body and limbs, to indenture proper position for sensor placement. Although we cannot exclude the possibility of slight movement, we specifically included only docile animals that wew accustomed to handling and excluded those that exhibited excessive movement. In this study, the use of the Nellcor Oxi Max adhesive finger sensor significantly increased the success rate of detecting reliable pulse rate values from 63% to 72.8%, compared to the Y-clip. The likely reason for this improvement is that the Nellcor Oxi Max probe applies less compression than the clip, which may facilitate better detection of pulsations. This highlights the importance of selecting an appropriate sensor type to optimize pulse oximetry performance, particularly in species where tissue fragility and perfusion variability may affect signal acquisition. A study on anesthetized dogs and cats [28] examined the effect of various probe configurations on SpO_2_ values, including the use of gauze swabs of different thickness between the tongue and the probe, red cotton fabric, and a sheet of white paper. Mair and colleagues [28] suggested that variations in contact pressure were primarily responsible for significant differences in SpO_2_ measurements. Different SpO_2_ values were associated with different perfusion index (PI) values, an index detected by the pulse oximeter to estimate peripheral perfusion at the probe site. Zanusso [29] also found that PI affected the performance of the CO pulse oximeter in anesthetized dogs, demonstrating the importance of perfusion in signal acquisition. The Edan VE-H100B does not provide a value for PI, so we cannot confirm a difference in the probe pressure between the Y-clip and the Nellcor Oxi Max adhesive finger sensor based on this index. The inability to measure PI with the Edan VE-H100B may have limited the assessment of perfusion-related factors that may have influenced success rate of the device. Future studies should include pulse oximeters capable of measuring PI to evaluate its role in signal acquisition and failure rates. Local perfusion at the application site may also be influenced by patient-related factors. In humans, peripheral vasoconstriction has been shown to decrease the accuracy of pulse oximetry [30]. In cats, the failure rate of the pulse oximeter on the tongue was affected by vasoconstriction induced by dexmedetomidine [16]. In our study, all rabbits were docile and healthy and accustomed to human handling, making high vasoconstriction due to manipulation stress unlikely. Additionally, all rabbits were examined in the same room, with no other animals present, to ensure a controlled environment. However, slightly varying levels of peripheral perfusion may have influenced the success rate of the devices. Notably, the Edan VE-H100B device failed to read SpO_2_ values in two of the 14 animals where the Masimo Rad-5 also failed. It is possible that local perfusion was similarly modified in these subjects. However, the Masimo Rad-5 has been shown to be less affected by vasoconstriction than the Edan VE-H100B [16]. In sheep, the use of Masimo SET technology, as used in the present study, resulted in a 0% failure rate even under conditions such as hypotension, poor perfusion, and anemia [31]. Besides local perfusion, the anatomical conformation of the probe site in some rabbits may have also contributed to the failure to detect SpO_2_ values. Both the width and the conformation of the anatomical site can affect the alignment between the light-emitting diode (LED) and the detector of the probe. In a study in anesthetized dogs and cats, SpO_2_ values were strongly influenced by tongue thickness and slightly influenced by the intersection angle of the Y-clip on the tongue [32]. Moreover, it is crucial for optimal performance of pulse oximeter sensors that the sensor closely conforms to the contours of the tissue being evaluated. Additionally, keeping the LED and photodetector on opposite sides of the tissue as parallel as possible is essential for proper sensor function. In our study, the sensor with flexible LED and photodetector sides ensured conformance to the contour of the site, while the Y-clip, likely due to its rigidity, was less underperforming [33,34].

The success rate in this study varied depending on the location of the probe, but it was lower for all devices on the tail compared to the forelimb. This discrepancy may be due to the slight compression of the probe on the tail, which may be thinner than the forelimb. Future studies could further validate this hypothesis by measuring local perfusion, for example, using PI. Excessive compression of the auricular vasculature by the clamp holding the probe can result in a poor signal in rabbits [9]. Again, the greater pressure exerted by the Y-clip compared to the Nellcor Oxi Max adhesive finger sensor may explain why the success rate for pulse rate on the tail was 48% with the Y-clip and 60.8% with the Nellcor Oxi Max adhesive finger sensor.

The study results suggest that the forelimb may be a more reliable site for obtaining accurate SpO_2_ and pulse rate values using the Masimo Rad-5 and the Edan VE-H100B with Y-clip. This is supported by the smaller mean bias between the clinical heart rate and detected pulse rate with both devices on the forelimb compared to the tail. When using the Edan VE-H100B with Nellcor Oxi Max adhesive finger probe on the forelimb, the limits of agreement between the detected pulse rate and the clinical heart rate ranged from -26–19 bpm. Considering that the normal heart rate in rabbits is 217.3 ± 21.5 bpm and their normal heart rate variability [35], a difference of 26 bpm may be not clinically significant. In contrast, in species such as cats, which have lower heart rates than rabbits, even small differences may be significant. In one study in cats, failure of both pulse oximeters was defined as the pulse rate that differed by more than 10 bpm from the electrocardiogram (ECG) heart rate [16].

In our study, inaccuracies in clinical measurement of heart rate cannot be completely ruled out. However, in our awake rabbits, monitoring heart rate using more accurate instruments, such as ECG, was not feasible because of the stress it would cause to the animals and the potential for movement artifacts in ECG recording [36].

Moreover, the accuracy of SpO_2_ was not evaluated by measuring arterial oxygen saturation (SaO_2_) in our study because arterial blood sampling was deemed unethical and there was no clinical reason to take arterial blood. In humans, most pulse oximeters manufacturers claim an accuracy of 2%, which is the standard deviation of the differences between SpO_2_ and SaO_2_ [37]. Although we did not measure SaO_2_, we established a fairly strict definition of success rate in our study. Successful measurements were defined not only by the detection of SpO_2_ but also by whether the value was clinically reliable. Thus, by restricting the definition of success, we limited the inclusion of very inaccurate SpO_2_ measurements. Moreover, in our study, we also considered that the color of the mucous membranes was pink, although the color of the mucous membranes may not be an extremely accurate way to assess oxygenation status.

Future studies could evaluate the performance of the devices used in this study under both awake and anesthetized conditions, as anesthesia-related complications are more common in rabbits. Sedative and anesthetic drugs may increase the failure rate of pulse oximetry, as observed in cats, where the Edan device showed a higher failure rate than the Masimo under anesthesia [16,38]. However, the reduced presence of motion artifacts in anesthetized animals may improve the device performance. In a study in dogs, the ability of SpO_2_ to serve as a surrogate for PaO_2_ was better in anesthetized patients compared to awake patients [39]. Additionally, since the Masimo SET technology has demonstrated greater performance in pulse rate detection under conditions of motion, dark pigmentation, and low perfusion, its use in anesthetized rabbits could be further explored. Moreover, the ability of the devices used in this study to accurately detect SpO_2_ values should be tested over a wider range of SpO_2_ levels. Notably, a significant overestimation of SpO_2_ has been reported in newborn lambs when SpO_2_ was below 70% [14]. Additionally, future studies could compare the effectiveness of reflectance probes versus traditional transmission probes in pulse oximetry, especially in rabbits. Reflectance probes may provide more accurate measurements in areas of low blood flow or smaller sites, such as the ear or tongue, and may help assess factors that affect SpO₂ accuracy.

## Conclusions

In our study, the Edan VE-H100B had a significantly higher success rate in detecting SpO_2_ and pulse rate than the Masimo Rad-5 in awake rabbits. The forelimb was the more reliable probe site than the tail, and the Nellcor Oxi Max adhesive finger probe improved measurement reliability. These results underscore the critical impact of device selection and probe placement on pulse oximetry in rabbits.
